# Genomes and Transcriptomes of Partners in Plant-Fungal- Interactions between Canola (*Brassica napus)* and Two *Leptosphaeria* Species

**DOI:** 10.1371/journal.pone.0103098

**Published:** 2014-07-28

**Authors:** Rohan G. T. Lowe, Andrew Cassin, Jonathan Grandaubert, Bethany L. Clark, Angela P. Van de Wouw, Thierry Rouxel, Barbara J. Howlett

**Affiliations:** 1 School of Botany, The University of Melbourne, Parkville, Victoria, Australia; 2 ARC Centre of Excellence in Plant Cell Walls, School of Botany, The University of Melbourne, Parkville, Victoria, Australia; 3 INRA-Bioger, UR1290, Thiverval-Grignon, France; University of Nebraska-Lincoln, United States of America

## Abstract

*Leptosphaeria maculans* ‘brassicae’ is a damaging fungal pathogen of canola (*Brassica napus*), causing lesions on cotyledons and leaves, and cankers on the lower stem. A related species, *L. biglobosa* ‘canadensis’, colonises cotyledons but causes few stem cankers. We describe the complement of genes encoding carbohydrate-active enzymes (CAZys) and peptidases of these fungi, as well as of four related plant pathogens. We also report dual-organism RNA-seq transcriptomes of these two *Leptosphaeria* species and *B. napus* during disease. During the first seven days of infection *L. biglobosa* ‘canadensis’, a necrotroph, expressed more cell wall degrading genes than *L. maculans* ‘brassicae’, a hemi-biotroph. *L. maculans* ‘brassicae’ expressed many genes in the Carbohydrate Binding Module class of CAZy, particularly CBM50 genes, with potential roles in the evasion of basal innate immunity in the host plant. At this time, three avirulence genes were amongst the top 20 most highly upregulated *L. maculans* ‘brassicae’ genes *in planta*. The two fungi had a similar number of peptidase genes, and trypsin was transcribed at high levels by both fungi early in infection. *L. biglobosa* ‘canadensis’ infection activated the jasmonic acid and salicylic acid defence pathways in *B. napus*, consistent with defence against necrotrophs. *L. maculans* ‘brassicae’ triggered a high level of expression of isochorismate synthase 1, a reporter for salicylic acid signalling. *L. biglobosa* ‘canadensis’ infection triggered coordinated shutdown of photosynthesis genes, and a concomitant increase in transcription of cell wall remodelling genes of the host plant. Expression of particular classes of CAZy genes and the triggering of host defence and particular metabolic pathways are consistent with the necrotrophic lifestyle of *L. biglobosa* ‘canadensis’, and the hemibiotrophic life style of *L. maculans* ‘brassicae’.

## Introduction

As more fungal genome sequences become available, it is apparent that their complement of genes and transcriptomes reflects fungal lifestyles. Lifestyles of plant pathogenic fungi are often classified into three broad categories: biotrophy, where the pathogen feeds from live host cells, necrotrophy, where the host cells are killed ahead of colonisation, and hemi-biotrophy, where the pathogen feeds from living cells before switching to a necrotrophic style of growth. These designations are imprecise and as the mechanisms of pathogenicity in a range of fungi are elucidated, lifestyle boundaries become more blurred [1].

The fungal genus *Leptosphaeria* belongs to the class Dothideomycetes, which includes a number of economically important plant pathogens that have a range of lifestyles on their hosts [2]. The *Leptosphaeria* species complex has two species *L. maculans* and *L. biglobosa* [3] and several sub-species or clades including *L. maculans* ‘brassicae’ and ‘lepidii’, and *L. biglobosa* ‘canadensis’, ‘brassicae’, ‘australensis’ and ‘occiaustralensis’ [4]. The nomenclature for these fungi is currently under review [5]. *L. maculans* ‘brassicae’, a hemibiotroph, causes blackleg, the most important disease of *Brassica napus* (canola) worldwide. Airborne sexual spores (ascospores) released from infested crop residues from the previous year’s crop land on seedlings. Hyphae of germinated spores enter plant tissue via stomatal apertures and asymptomatically colonise the apoplastic spaces, between the plant cells. After eight to ten days, plant cells collapse and asexual sporulation begins within the necrotic leaf lesion. Hyphae then grow along the petiole and the stem, often resulting in a canker that girdles the stem causing lodging of the plant [6]. In contrast, *L. biglobosa* ‘canadensis’ is not well-characterised. Although it causes cotyledonary lesions, stem cankers are rarely produced [7].

*Leptosphaeria maculans* ‘brassicae’ has numerous ‘gene for gene’ interactions with *B. napus* whereby an avirulence allele in the fungus renders it unable to attack cultivars with the corresponding resistance gene. This ‘gene for gene’ resistance is expressed in seedlings; five avirulence genes have been cloned so far - *AvrLm1*, *AvrLm4-7*, *AvrLm6 AvrLm1* and *AvrLmJ1* [8]–[12]. Only one resistance gene, *LepR3*, has been cloned from *B. napus* [13].

During infection fungi derive nutrition from the host plant, often by enzymatic degradation of proteins and carbohydrates. These latter enzymes are classed as Carbohydrate-Active enZymes (CAZys) and they often have well characterised domains. As well as providing nutrition, CAZy activity releases cell wall products that can act as DAMPs (Damage Associated Molecular Patterns) that activate the host immune system [14]. The biotrophic plant pathogens *Blumeria graminis*, *Puccinia graminis, Melampsora laricus-populina*, *Hyaloperonospora arabidopsis* and *Ustilago maydis* have fewer CAZy genes than necrotrophic plant pathogens do, perhaps because biotrophs do not need to digest plant cell walls for nutrition and they must evade the host immune system [15]–[17].

Genomic sequences are now available for many Dothideomycetes and an extensive comparative analysis of 18 of them, including *L. maculans* ‘brassicae’ has been published [18]. These include the *Brassica*-infecting pathogen, *Alternaria brassicicola*, as well as three wheat-infecting pathogens, *Stagonospora nodorum*, *Pyrenophora tritici-repentis*, and *Zymoseptoria tritici*. The former three fungi, like many members of the order Pleosporales, have a necrotrophic lifestyle releasing toxins soon after invasion, whilst *Zymoseptoria tritici,* like most members of the order Capnodiales, is a hemibiotroph, with an extended period as a biotroph before causing necrosis [19]–[21].

The *L. maculans* ‘brassicae’ genome is compartmentalised into AT-rich -that are gene-poor comprising up to 35% of the genome, and gene- rich regions that have a high GC content [2], while the genome of *L. biglobosa* ‘canadensis’ is 30 Mb and lacks AT-rich regions (Grandaubert et al. manuscript submitted). A limited amount of oligo-array transcriptome data have been produced for *L. maculans* ‘brassicae’ during *in vitro* culture and infection of *B. napus* [11], but transcriptome data have not been reported for *L. biglobosa* or any closely related Brassica pathogen.

Patterns of global gene expression can be generated by RNA-seq, a technique that enables analysis of dual transcriptomes; for instance, during a plant pathogen interaction. Furthermore RNA-seq can be exploited to analyse non-model organisms for which genomic resources are not well developed [22]. Few dual transcriptomes for plants and pathogenic fungi have been reported. Two recent reports of dual RNA-seq analysis of fungal diseases are of rice blast [23] and target leaf spot of sorghum [24]. Here we describe the genomes and transcriptomes of *L. biglobosa* ‘canadensis’ and *L. maculans* ‘brassicae’ and canola (*Brassica napus*), during infection and *in vitro.* Our aim is to characterise genes that each pathogen uses to evade detection by the host, or to derive nutrition from the host, viz. the carbohydrate-active enzymes and peptidases. We also examine genes upregulated by the host during infection by each pathogen.

## Methods

### Fungal isolates and culture conditions

*L. maculans* ‘brassicae’ isolate IBCN18 and *L. biglobosa* ‘canadensis’ isolate 06J154, hereafter referred to as Lmb and Lbc, respectively, were subcultured on 10% Campbell’s V8 juice agar at 22°C with a 12 h light/12 h dark light cycle. Conidia (5×10^6^) were added to 30 mL of liquid medium and incubated in still culture in a petri dish (15 cm diameter) at 22°C in the dark. Culture media were either 10% Campbell’s V8 juice, or oilseed rape medium. The latter medium was prepared by homogenising leaves (200 g) of *B. napus* cv. Westar in a waring blender in a final volume of 1 L of water. The homogenate was centrifuged at 2000 *g* for 20 min and the resulting supernatant was filter sterilised (0.22 µm Millipore stericup filter).

### Plant growth and infection conditions

*Brassica napus* cv. Westar was used for all infection assays; it has no known resistance genes. Seedlings were grown in a glasshouse maintained at 25°C under natural lighting. Wounded cotyledons were infected with conidia (10 uL of a 1×10^5^ spores/mL suspension) or water (mock inoculum), at 10 days post sowing as described previously [25].

### Extraction of RNA and gene expression analysis

For RNA-seq analysis, *B. napus* cotyledons were infected with Lmb, Lbc or water (control mock inocula), and at 7 and 14 days post inoculation (dpi) tissue around the inoculation site was harvested using a cork borer (0.5 cm diameter) and then placed into liquid nitrogen before freeze drying and subsequent grinding under liquid nitrogen. Tissue samples were prepared in biological triplicate. RNA was extracted using Trizol reagent from infected tissue and from mycelia of Lmb and Lbc from 7-day still cultures grown in oilseed rape medium. RNA was then DNAase-treated (Life Technologies) and cleaned up.

The two biological replicates of each sample with the highest RNA integrity number values (>6) were sequenced with Illumina TruSeq version 3 chemistry on an Illumina HiSeq2000 sequencer at the Australian Genome Research Facility. *In vitro* derived RNA was sequenced with 100 bp paired-end reads in order to aid gene annotation, and *in planta* derived RNA was sequenced with 100 bp single-end reads. A total of 15.5 Gbp sequence was generated from the *in vitro* libraries of the two fungi (7.75 Gbp per sample), and 72 Gbp sequence was generated from 12 *in planta* libraries (6 Gbp per sample) (Table S1 in file S2). Reads were trimmed to a minimum phred quality score of 20 using Nesoni sequence software [26], orphaned members of pairs were retained, adaptor sequences were removed, and reads shorter than 20 bp were rejected. Trimmed reads were aligned to a reference genome sequence with Tophat v1.4.1 splice-junction mapper [26]. Reference genomes were Lmb isolate v23.1.3 [2], Lbc isolate J154 (Grandaubert et al., submitted), and a *Brassica* exon array curated unigene set representing 135,201 gene models from *B. napus*, *B. rapa* and *B. oleracea* [27]. Aligned reads were quantified using Cufflinks v1.0.3 transcript assembly and quantification software and denoted as average expression levels (FPKM - fragments per kilobase of exon per million mapped reads) [28]. Cufflinks was run with bias correction to reduce variance due to sequence positional bias during Illumina sequencing. Gene expression FPKM values are listed in the relevant supplementary tables. All of the aligned RNA-seq reads have been deposited at the NCBI Sequence Read Archive (SRA), accessible under bioproject accession PRJNA230885 or sequence read archive SRP035525. SRA files may be read using the NCBI SRA toolkit (http://www.ncbi.nlm.nih.gov/sra).

Quantitative RT-PCR experiments were carried out to determine levels of expression of genes containing CBM50 (LysM) domains. Cotyledon tissue from 32 *B. napus* cv. Westar seedlings was harvested at 3, 7 and 14 days after inoculation with Lmb isolate IBCN18. RNA (2 µg) was treated with DNase 1 (Life Technologies) for 1 h at 25°C, and reverse-transcribed using oligo-dT primer and Superscript III (Life Technologies) at 50°C for 1 h. Levels of gene expression were determined by qPCR using SensiMix (dT) SYBR Green PCR kit (Bioline) in a Corbett Rotor-Gene 3000 machine. Transcript levels of the gene of interest were normalized to that of Lmb actin as described previously (Gardiner et al., 2004). Primers are listed in Table S2 in file S2.

### Annotation of genes and domains

Genes encoding CAZys were identified in six dothideomycetes (Lmb, Lbc, *A. brassicicola*, *S. nodorum*, *P. tritici-repentis* and *Z. tritici*) using www.cazy.org [29] and the dbCAN v3.0 HMM-based CAZy annotation server (http://csbl.bmb.uga.edu/dbCAN/) [30]. The major CAZy classes are Polysaccharide Lyases (PL), Glycosyl Transferases (GT), Glycosyl Hydrolases (GH), Carbohydrate Esterases (CE), Carbohydrate Binding Modules (CBM) and Auxiliary Activities (AA). Secretion signal peptides were predicted using the SignalP 4.0 algorithm [31]. Pfam domains (Pfam A and B) were identified using profile hidden Markov models with HMMER3.0 [32] and Pfam_scan.pl software; the e value cut off was set to 1e-5 [33]. LysM-containing genes (with CBM50 domain) were examined in more detail. Predicted gene models and aligned RNA-seq reads were viewed with the IGV browser [34], predicted intron splice sites were verified and translation start sites were checked for congruence with observed RNA-seq transcript boundaries. They were initially identified by comparison to the Pfam database [33], and then aligned to ECP6, the well characterised LysM-containing protein from *Cladosporium fulvum* [35]. Other *Leptosphaeria* proteins with LysM motifs were identified and characterised as described in supporting information (Figure S1 in file S1).

Peptidase domains were identified by BlastP [36] comparison of predicted protein sequences to the MEROPS ‘peptidase database pepunit.lib dataset’ of database of peptidase and inhibitor units (http://merops.sanger.ac.uk) [37].

### Analysis of expression of fungal and *B. napus* genes

The 100 most highly up regulated genes of Lmb and Lbc at 7 and 14 dpi, compared to *in vitro* growth, were identified from the RNA-seq data (Table S 3–6 in file S2). Averages of quantile-normalised log10-transformed FPKM values were calculated for each dataset for direct comparison of CAZy gene expression between Lmb and Lbc. Genes containing a CBM, GH, PL or AA domain and predicted to be secreted were analysed further. The sum of the expression values of each CAZy gene across the three treatments (*in vitro*, 7 and 14 dpi) was determined and the top 100 genes were identified. Quantile-normalisation was applied so that expression values of genes of both fungi could be directly compared and log10-transformed FPKM values were graphed. For each treatment, a heat map based on expression values was generated.

*B. napus* defence genes, 9-cis-epoxycarotenoid dioxygenase 3 (NCED), 1-amino-cyclopropane-1-carboxylate synthase 2(ACS2), chitinase (CHI), hevein-like protein (HEL), isochorismate synthase 1 (ICS1), Pathogenesis related protein 1(PR1), WRKY transcription factor 70, and plant defensin 1 (PDF1-2) [38] were identified (Table 1). Their RNA-seq expression was analysed at 7 and 14 dpi in inoculated and uninoculated cotyledons. Additionally, expression of *Brassica* genes involved in metabolic pathways was compared at 7 dpi by either Lmb or Lbc, and then analysed using MapMan, software that processes large gene expression datasets into metabolic pathways or other processes [39]. A Wilcoxon rank sum test with Bonferroni correction for multiple tests was used within MapMan to identify 20 functional categories of *B. napus* genes that were significantly regulated in response to infection by Lmb or Lbc.

**Table 1:** *B. napus* defence genes analysed [50].

Gene	Full Name	Defence Signalling Pathway	*B. napus* RRES unigene ID	GenBank accession of CDS
NCED3	9-cis-epoxycarotenoid dioxygenase 3	Abscisic acid	rres040714.v1	EV137674
ACS2a	1-amino-cyclopropane-1-carboxylate synthase 2	Ethylene	rres079827.v1	HM450312
CHI	Chitinase	Ethylene/salicylic acid//jasmonic acid	rres036231.v1	X61488
HEL	Hevein-like protein	Ethylene/jasmonic acid	rres036743.v1	FG577475
ICS1	Isochorismate synthase 1	Salicylic acid	rres059693.v1	EV225528
PR-1	Pathogenesis related protein 1	Salicylic acid	rres112514.v1	BNU21849
WRKY70	WRKY transcription factor 70	Salicylic acid	rres038189.v1	EV113862
PDF1.2	Plant Defensin 1.2	Jasmonic acid	rres071321.v1	KC967203

Defence signalling pathways indicate regulatory pathway to which the gene belongs. RRES unigenes are listed from the Brassica exon array [27], to which RNA-seq reads were aligned. Corresponding GenBank accession numbers are given for each gene. The response of each gene (with the exception of PDF1.2) to its corresponding signalling pathway was confirmed by Sasek et al [50].

## Results and Discussion

### Symptoms and lifestyles of *L. biglobosa* ‘canadensis’ and *L. maculans* ‘brassicae’ on cotyledons of *B. napus*

**Figure 1:**
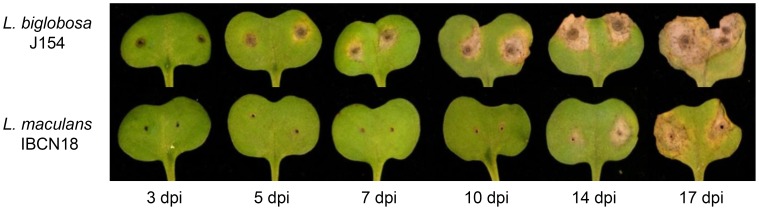
Symptoms on cotyledons of *B. napus* cv. Westar infected with *L. maculans* ‘brassicae’ or *L. biglobosa* ‘canadensis’. *B. napus* cv. Westar cotyledons were wounded and inoculated with Lmb or Lbc spores and disease allowed to progress for 17 days post inoculation (dpi). Cotyledons were harvested and photographed at 3, 5, 7, 10, 14, and 17 dpi to track lesion development by the two pathogens.

*Leptosphaeria maculans* ‘brassicae’ (Lmb) and *L. biglobosa* ‘canadensis’ (Lbc) exhibited different timing of symptom development on *B. napus* cotyledons (Figure 1). Lmb had a visually asymptomatic phase until after 7 days post inoculation (dpi), when lesions became visible. Lbc produced initial darkening of the cotyledon tissue at 3 dpi, and cell death and necrosis were apparent by 5 dpi and lesions increased in size until 17 dpi. At this time lesions caused by Lmb were of a similar size. Thus Lmb at 7 dpi appeared to be growing biotrophically, but at 14 dpi was growing necrotrophically, whilst Lbc was necrotrophic from 5 days onwards, although as previously described, its growth is usually arrested before it can colonise the stem [7]. The rapid *in planta* necrosis caused by Lbc was similar to that previously described for *L. biglobosa* ‘brassicae’ [40].

### General features of fungal and plant transcriptomes

**Figure 2:**
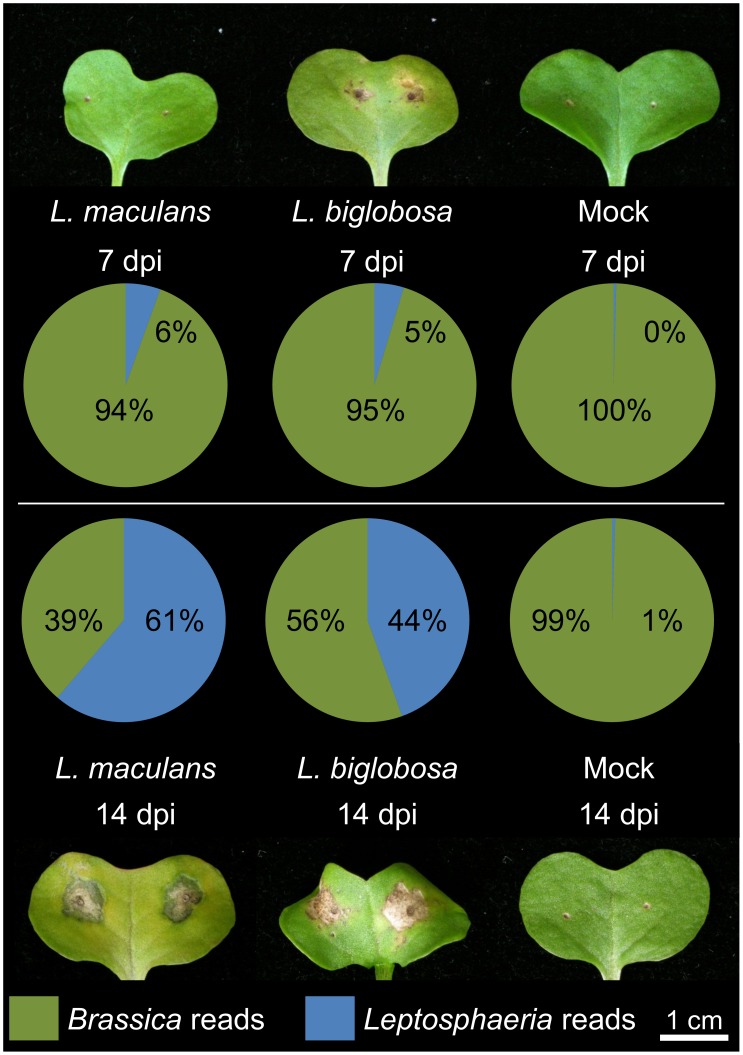
Percentages of plant and fungal transcripts in *B. napus* cotyledons, uninoculated or at 7 and 14 days post-inoculation dpi with *L. maculans* ‘brassicae’ (Lmb) or *L. biglobosa* ‘canadensis’ (Lbc). *B. napus* cotyledons were infected with Lmb, Lbc or water (control mock inoculum). Total RNA was extracted from lesion tissue at 7 and 14 dpi for Illumina RNA-seq sequencing. The percentage of reads aligned to the Brassica exon array unigene set (green) or the reference genomes for Lmb or Lbc (blue) is presented, as well as a photo of each representative infection. All of the aligned sequence reads were deposited at the NCBI Sequence Read Archive (SRA), accessible under bioproject accession SRP035525.

**Figure 3:**
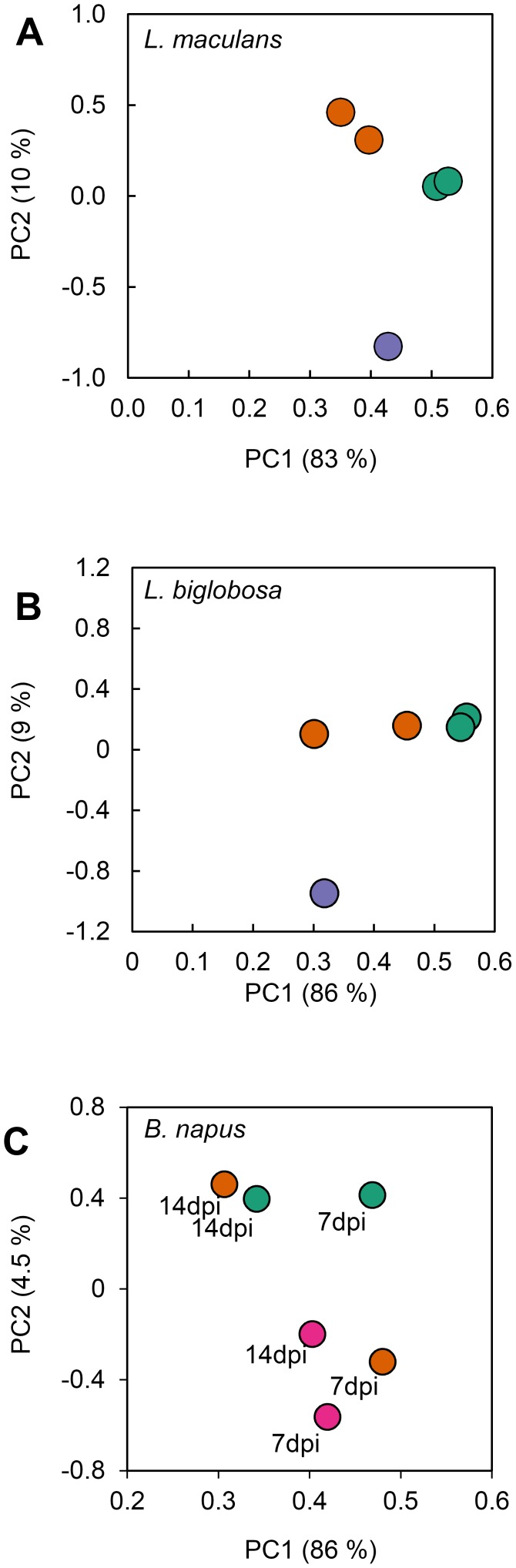
Principal Component Analyses of duplicate sets of RNA-seq data for *L. maculans* ‘brassicae’ (Lmb), *L. biglobosa *‘canadensis’ (Lbc) and *B. napus*. Scores plots for principal component analysis of expression values (FPKM) of genes of Lmb IBCN18 (A), Lbc J154 (B), or *B. napus* (C). Gene expression values during infections of cotyledons are plotted in orange (7 dpi) and green (14 dpi). Gene expression values during growth *in vitro* are plotted in purple; those in mock-infected cotyledons are plotted in magenta. The percentage of the total variance explained is listed on each axis label.

RNA-seq was used to define the transcriptomes of both pathogens and host during infection. A total of 87.5 Gbp of raw sequence was generated across all libraries (Table S1 in file S2). In spite of the differences in disease symptoms at 7 dpi, the percentage of Lmb and Lbc reads aligned to the Lmb and Lbc reference genomes was similar (5 and 6% of the total), while 61 and 44% of reads aligned to the reference genomes at 14 dpi (Figure 2). These data reflect that by 14 dpi the plant tissue is heavily colonised by both fungi. As expected, very few reads (less than 1%) from the mock-infected plant libraries aligned to the *Leptosphaeria* reference genomes. These libraries represent highly complex large datasets and Principal Component Analysis was carried out to compare the overall features of the transcriptomes, particularly gene identity and expression level. FPKM values were calculated for each gene in the three organisms. The duplicate sets of RNA-seq data were very similar, with the exception of the data for Lbc at 7 dpi. Gene expression of Lmb in the three conditions (7 and 14 dpi, and *in vitro* growth) was clearly distinguishable one from another (Figure 3), and expression profiles of both fungi *in vitro* were more distinctive than *in planta*. Most of the variance (around 80%) was captured in PC1 for Lmb (Figure 3A) and Lbc infections (Figure 3B), where the 7 and 14 dpi time points were distinguished from each other. The difference between *in vitro* and *in planta* samples was captured in PC2 in for both Lmb and Lbc plots. *B. napus* gene expression was markedly different at 7 dpi after infection by Lmb compared to Lbc, but similar at 14 dpi. The response of *B. napus* to Lmb at 7 dpi was similar to that of mock inoculation, implying that at this time the plant had not mounted a strong response to infection (Figure 3C).

Since microarray expression (Nimblegen) data for another isolate of Lmb (v23.1.3) were available [2], the relative levels of expression of three avirulence genes (*AvrLm1*, *AvrLm4-7* and *AvrLm11*), present in both Lmb isolates were compared to check for broad agreement between the two technologies (microarrays and RNA-seq). These avirulence genes were ranked in the top 100 most highly expressed genes *in planta* for both microarray and RNA-seq, and both techniques reported a top 10 ranking for *AvrLm1* and *AvrLm4-7* (see below).

Only eight of the 20 most highly upregulated *in planta* genes of Lmb had Pfam domains (Table 2). These included one gene with five CBM50 (LysM) domains (see later); other genes included three cytochrome P450 monooxygenases, and a transferase present in a gene cluster containing a polyketide synthase. Several hypotheticals were amongst the top 20 genes, as well as three avirulence genes, *AvrLm1, AvrLm4-7* and *AvrLmJ1* [12]. In contrast, 19 of the 20 most highly upregulated *in planta* genes of Lbc had Pfam domains (Table 3), although five of these were conserved domains without functional annotation (Pfam-Bs, or Domain of Unknown Function (DUF)). Two cellulases, four other glycosyl hydrolases, as well as three peptidases were present.

**Table 2:** Top 20 upregulated genes of *L. maculans* ‘brassicae’ isolate IBCN18 seven days after inoculation of *B. napus* cv. Westar.

Gene ID	Gene name	7dpi *in planta* (FPKM)	14dpi *in planta* (FPKM)	*in vitro* (FPKM)	Log2 (7dpi FPKM/in vitro FPKM)	Pfam description and accession	Pfam expect score
Lema_P114790.1		12645.1	1163.5	0.1	16.9		
Lema_P114800.1		4066.8	728.9	0.1	15.3		
Lema_P092260.1		1375.5	30.2	0.1	13.7		
Lema_P086290.1	*AvrLm1*	1831.5	38.6	0.1	13.7		
Lema_P049660.1	*AvrLm4-7*	2540.0	70.8	0.3	13.1		
Lema_uP037480.1		793.8	42.8	0.1	13.0		
Lema_P084480.1		2645.1	207.0	0.5	12.5	Pfam-B_14615 [PB014615]	4.2E-08
Lema_P054900.1		567.4	24.7	0.1	12.5		
Lema_uP070880.1	*AvrLmJ1*	1349.9	59.3	0.3	12.4		
Lema_uP082260.1		521.8	3.4	0.1	12.3		
Lema_P087720.1		498.9	3.9	0.1	12.3	Cytochrome P450 [PF00067.17]	9.0E-57
Lema_P070100.1	*Lm5LysM*	2672.9	105.4	0.8	11.7	LysM domain [PF01476.15]	3.9E-08
Lema_uP121480.1		295.4	19.0	0.1	11.5		
Lema_P006160.1		5011.4	1356.8	1.8	11.5	Pfam-B_2613 [PB002613]	2.1E-33
Lema_P087710.1		282.5	6.2	0.1	11.5	Transferase family [PF02458.10]	5.7E-23
Lema_P087700.1		279.5	2.1	0.1	11.4	Cytochrome P450 [PF00067.17]	6.4E-57
Lema_uP002340.1		1624.1	96.0	0.7	11.2		
Lema_P087750.1		223.7	2.1	0.1	11.1	Cytochrome P450 [PF00067.17]	4.1E-43
Lema_P037680.1		1289.1	6.9	0.6	11.0	Pfam-B_8517 [PB008517]	8.0E-140
Lema_uP123070.1		388.9	34.8	0.2	10.9		

The top 100 *in planta* upregulated genes are listed in Table S3 in file S2.

**Table 3:** Top 20 upregulated genes of *L. biglobosa* ‘canadensis’ isolate J154 seven days after inoculation of *B. napus* cv. Westar.

Gene name	Gene name	7dpi in planta (FPKM)	14dpi in planta (FPKM)	*in vitro* (FPKM)	Log2 (7dpi FPKM/in vitro FPKM)	Pfam description and accession	Pfam expect score
Lb_j154_P000524		886.3	18.0	0.01	17.0	Protein of unknown function (DUF3678) [PF12435.3]	1.20E-01
Lb_j154_P003557		515.7	63.8	0.01	16.2	Pfam-B_18451 [PB018451]	3.60E-69
Lb_j154_P001652		1083.8	151.2	0.1	13.5	Cellulase (glycosyl hydrolase family 5) [PF00150.13]	7.70E-20
Lb_j154_P009247		1012.1	146.1	0.1	13.2	Glycosyl hydrolases family 12 [PF01670.11]	1.20E-31
Lb_j154_P006347		341.8	76.1	0.04	13.2	Deuterolysin metalloprotease (M35) family [PF02102.10]	5.00E-86
Lb_j154_P002168		878.3	9.0	0.1	12.8	Endoribonuclease L-PSP [PF01042.16]	7.30E-11
Lb_j154_P001276		317.1	24.8	0.05	12.6	Pfam-B_19830 [PB019830]	2.60E-36
Lb_j154_P001225		605.1	114.3	0.5	10.4	Putative cyclase [PF04199.8]	2.00E-13
Lb_j154_P010735		5991.3	6460.7	4.7	10.3	Alcohol dehydrogenase GroES-like domain [PF08240.7]	1.60E-23
Lb_j154_P008673		1467.7	220.5	1.2	10.3	Pfam-B_19830 [PB019830]	1.10E-38
Lb_j154_P004138		7216.7	1370.4	6.0	10.2	Trypsin [PF00089.21]	6.60E-64
Lb_j154_P004093		3826.7	456.8	3.2	10.2	Glycosyl hydrolases family 39 [PF01229.12]	3.20E-05
Lb_j154_P006834		410.0	148.0	0.4	10.1		
Lb_j154_P005286		953.8	53.9	0.9	10.0	Pectate_lyase [PF03211.8]	1.50E-58
Lb_j154_P001719		1410.8	291.4	1.4	9.9	Trypsin [PF00089.21]	7.90E-66
Lb_j154_P000527		12641.8	8785.2	13.1	9.9	Pfam-B_14072 [PB014072]	4.30E-34
Lb_j154_P009204		694.9	99.8	0.7	9.9	Cellulase (glycosyl hydrolase family 5) [PF00150.13]	6.00E-13
Lb_j154_P006775		307.5	11.8	0.4	9.7	Putative amidotransferase (DUF4066) [PF13278.1]	1.10E-26
Lb_j154_P001246		1340.6	88.9	1.9	9.5	Glycosyl hydrolases family 43 [PF04616.9]	1.20E-59
Lb_j154_P001995		617.0	66.2	0.9	9.4	Sugar (and other) transporter [PF00083.19]	1.10E-95

The top 100 *in planta* up regulated genes are listed in Table S4 in file S2.

At 14 dpi, 14 of the top 20 *in planta*-expressed genes of Lmb were hypothetical genes lacking any functional annotation (Table 4). Few were also in the top 20 at 7 dpi, with the exception of *AvrLm1* and LemaP114790.1, which has no Pfam domains and was the most highly expressed gene at both time points. Even though there was a high degree of necrosis on cotyledons at 14 dpi, only two hydrolytic enzymes (trypsin and a glycohydrolase 7) were included in the top 20 most highly expressed Lmb genes. Two of the top 20 genes of Lbc were CAZys; two peptidases and three dehydrogenases, genes that may have degrading roles were also present (Table 5).

**Table 4:** Top 20 upregulated genes of *L. maculans* ‘brassicae’ isolate IBCN 18 fourteen days after inoculation of *B. napus* cv. Westar.

Gene ID	Gene name	7dpi in planta (FPKM)	14dpi in planta (FPKM)	*in vitro* (FPKM)	Log2 (14dpi FPKM/in vitro FPKM)	Pfam description and accession	Pfam expect score
Lema_P114790.1		12645.1	1163.5	0.1	13.51		
Lema_P114800.1		4066.8	728.9	0.1	12.83		
Lema_P082270.1		861.2	3339.4	0.9	11.86	Trypsin [PF00089.21]	3.30E-66
Lema_P043000.1		106.6	762.8	0.7	10.12	Pfam-B_11894 [PB011894]	3.90E-35
Lema_P006160.1		5011.4	1356.8	1.8	9.59	Pfam-B_2613 [PB002613]	2.10E-33
Lema_P110730.1		3.4	66.5	0.1	9.38	Flavin-containing amine oxidoreductase [PF01593.19]	6.90E-27
Lema_uP085940.1		22.6	56.5	0.1	9.14		
Lema_P085920.1		55.2	74.6	0.1	8.97		
Lema_P084480.1		2645.1	207.0	0.5	8.84	Pfam-B_14615 [PB014615]	4.20E-08
Lema_P117020.1		211.3	85.9	0.2	8.81	Pfam-B_7042 [PB007042]	6.50E-17
Lema_uP037480.1		793.8	42.8	0.1	8.74		
Lema_P077490.1		19.1	45.9	0.1	8.74	Cutinase [PF01083.17]	8.00E-49
Lema_P006340.1		14.3	249.7	0.6	8.71	Sugar (and other) transporter [PF00083.19]	2.70E-88
Lema_P060160.1		191.1	60.0	0.1	8.71		
Lema_P036670.1		113.9	213.6	0.6	8.58	Endoribonuclease L-PSP [PF01042.16]	1.10E-10
Lema_P013700.1		515.5	628.0	1.9	8.35	Glycoside hydrolase family 7 [PF00840.15]	3.30E-190
Lema_P085930.1		24.6	88.9	0.3	8.33		
Lema_P092260.1		1375.5	30.2	0.1	8.24		
Lema_P077180.1		24.1	30.2	0.1	8.24		
Lema_P086290.1	AvrLm1	1831.5	38.6	0.1	8.17		

The top 100 *in planta* up regulated genes are listed in Table S5 in file S2.

**Table 5:** Top 20 upregulated genes of *L. biglobosa* ‘canadensis’ isolate J154 fourteen days after inoculation of *B. napus* cv. Westar.

Gene ID	Gene name	7dpi in planta (FPKM)	14dpi in planta (FPKM)	*in vitro* (FPKM)	Log2 (14dpi FPKM/in vitro FPKM)	Pfam description and accession	Pfam expect score
Lb_j154_P003075		156.1	116.5	0.01	14.0		
Lb_j154_P003557		515.7	63.8	0.01	13.2	Pfam-B_18451 [PB018451]	3.6E-69
Lb_j154_P000524		886.3	18.0	0.01	11.3	Protein of unknown function (DUF3678) [PF12435.3]	1.2E-01
Lb_j154_P006347		341.8	76.1	0.04	11.0	Deuterolysin metalloprotease (M35) family [PF02102.10]	5.0E-86
Lb_j154_P001652		1083.8	151.2	0.1	10.6	Cellulase (glycosyl hydrolase family 5) [PF00150.13]	7.7E-20
Lb_j154_P009247		1012.1	146.1	0.1	10.4	Glycosyl hydrolase family 12 [PF01670.11]	1.2E-31
Lb_j154_P010735		5991.3	6460.7	4.7	10.4	Alcohol dehydrogenase GroES-like domain [PF08240.7]	1.6E-23
Lb_j154_P001707		63.1	9.2	0.01	10.4		
Lb_j154_P006327		113.6	8.5	0.01	10.2		
Lb_j154_P008556		1.4	4.9	0.01	9.4	Mediator complex subunit 27 [PF11571.3]	1.8E-01
Lb_j154_P000527		12641.8	8785.2	13.1	9.4	Pfam-B_14072 [PB014072]	4.3E-34
Lb_j154_P000350		32.9	4.3	0.01	9.3		
Lb_j154_P006232		3710.7	5194.8	8.5	9.2		
Lb_j154_P005193		1399.2	1588.4	2.8	9.2		
Lb_j154_P003830		27.8	38.5	0.1	9.1	Serine carboxypeptidase [PF00450.17]	4.2E-87
Lb_j154_P009122		5.3	3.5	0.01	9.0		
Lb_j154_P003076		158.0	118.9	0.2	9.0	Uncharacterized protein conserved in bacteria (DUF2321) [PF10083.4]	2.9E-01
Lb_j154_P001276		317.1	24.8	0.05	9.0	Pfam-B_19830 [PB019830]	2.6E-36
Lb_j154_P008991		3272.4	3034.9	6.1	9.0	Short-chain dehydrogenase [PF00106.20]	9.0E-27
Lb_j154_P003555		1776.8	1658.6	3.5	8.9	Alcohol dehydrogenase GroES-like domain [PF08240.7]	2.2E-22

The top 100 *in planta* up regulated genes are listed in Table S6 in file S2

Small secreted proteins (SSPs) were well represented in the most highly in planta upregulated genes of Lmb. These included hypothetical, avirulence and CAZy genes. Ohm et al (2012) compared the small secreted proteins of 18 Dothideomycete fungi including Lmb isolate 23.1.3, and found that 21.3% of all Lmb SSPs identified were singletons with no homologue in any of the other genomes [18]. A recent study found 30 of the 100 most highly expressed SSPs *in planta* at 7 dpi were unique to Lmb [52].

In summary, amongst the top 20 most highly expressed genes *in planta*, fewer encoding cell wall degrading enzymes were expressed by Lmb than by Lbc at 7 and 14 dpi. Cell wall degrading enzymes not only provide nutrition for the pathogen by hydrolysing carbohydrates, but facilitate fungal progression through the plant apoplast (intercellular spaces), during biotrophic stages of infection. The repertoire and expression of fungal CAZy genes presumably reflects the cognate carbohydrate present in the host plant and it is notable that enzymes such as those degrading cellulose are very highly expressed. The cell wall of leaves of *Arabidopsis thaliana*, which like *B. napus* is a crucifer, contain polysaccharides including rhamnogalacturonan I and II (as pectin), xyloglucan, glucuronoarabinoxylan, and cellulose (14%). Presumably the cotyledon has a similar polysaccharide profile. An additional 14% of the wall is composed of protein [41]. More than a third of the carbohydrate is soluble in phosphate buffered saline, including half of the total pectin, suggesting it would be readily available to a pathogen growing in the apoplast [41].

### Distribution of CAZy domains and their expression

In view of the dominance of CAZys in the 20 most highly expressed genes of Lbc after seven days *in planta*, CAZy domains were sought in genome sequences of Lbc, Lmb, and also in four other Dothideomycetes for comparison. The repertoire of domains derived from the dbCAN database and its HMM-based sequence similarity search was consistent with CAZy classifications carried out previously on Dothideomycetes (Table S7 in file S2) [18], [42], and the www.cazy.org annotation for Lmb isolate v23.1.3. In general, the numbers of carbohydrate-binding module (CBM), glycosyl hydrolase (GH), glycosyl transferase (GT), and polysaccharide lyase (PL) domains were in agreement with previous studies (Table 6). CAZys are divided into sub classes, generally based on substrate specificity or ligand; some of these are described later. Two CAZy classes in Lmb were identified more frequently using our analyses; CE domains (109 vs 34) and AA (78 vs 27). The difference in numbers of CE domains was probably because 27 CE1 and 42 CE10 domains had been previously excluded from the CAZy expert curated dataset on the basis that they were likely to act upon non-carbohydrate substrates [43]. AA domains are a newly formed CAZy classification for ligninolytic or lytic polysaccharide monooxygenases. Our automated approach may have included closely related monooxygenases that do not degrade lignin or polysaccharides.

**Table 6:** Distribution of CAZy domains in predicted proteins of six Dothideomycetes.

Number of domains							
CAZy domain	AA	CBM	CE	GH	GT	PL	Total
Fungus (lifestyle)							
*Leptosphaeria biglobosa* (necrotroph)	98	85	117	234	106	23	663
*Leptosphaeria maculans* (hemibiotroph)	78	63	109	217	100	19	586
*Alternaria brassicicola* (necrotroph)	92	66	120	233	92	24	627
*Stagonospora nodorum* (necrotroph)	122	66	143	267	96	10	704
*Pyrenophora tritici-repentis* (necrotroph)	114	56	124	246	105	10	655
*Zymoseptoria tritici* (hemibiotroph)	55	26	97	199	108	4	489

The classes of CAZy domains are Auxiliary Activities (AA) Carbohydrate Binding Modules (CBM), Carbohydrate Esterases (CE), Glycosyl Hydrolases (GH), Glycosyl Tranferases (GT) Polysaccharide lyases (PL). Some genes contain multiple CAZy domains.

Most of the CAZy containing-genes had only one CAZy domain, but several had multiple CBM domains, particularly of the subclass CBM50 (see later and Figure S1 in file S1). Five of the six dothideomycetes had between 580 and 700 CAZy domains, but *Z. tritici* only had 489, which is in general agreement with previous findings [44]. Overall the two *Leptosphaeria* species had similar complements of CAZy genes. *Alternaria brassicicola* had the next most similar profile. *Pyrenophora tritici-repentis* had a profile more similar to *Stagonospora nodorum* than to *Leptosphaeria*, consistent with the host of the two former fungi being a monocotyledonous cereal, rather than a dicotyledonous oilseed plant. Dicot cell walls generally contain a higher proportion of pectin, compared to glucuronoarabinoxylan, which is predominant in a typical monocot cell wall and believed to partially substitute for low levels of pectin in monocot cell walls [15]. *Zymoseptoria tritici* had a distinct profile with many fewer CAZy genes. This is congruent with previously reported characteristics of the members of order Capnodiales, versus the Pleosporales, to which the other five fungi belong. *L maculans* ‘brassicae’ had fewer AA domains than Lbc, *S. nodorum* and *P. tritici-repentis* did. The number of PL domains ranged from 19 to 24 in Lmb, Lbc and *A. brassicicola*. In contrast, the three wheat pathogens, *S. nodorum, P. tritici repentis* and *Z. tritici* had many fewer – between 4 and 10.

Although the complement of CAZys in the two *Leptosphaeria* species was generally similar, there were some interesting differences. The major difference was in the CBM class with >30% more domains present in Lbc than in Lmb. *L maculans* ‘brassicae’ had fewer CBM18-containing-genes than Lbc, but a similar number of CBM18 domains. CBM18 domains in Lmb were in a more compact gene structure (28 domains in 11 genes); indeed this domain was always present in multiple copies within a gene, five genes contained homopolymers of only CBM18. *L. biglobosa* ‘canadensis’ had 32 domains in 21 genes, eight of which had a single CBM18 domain. CBM50 (LysM), CE4, and GH18 CAZy domains occurred frequently in genes that had CBM18 domains. (Table S8 in file S2). In eukaryotes this domain often has a chitin-binding function and modules are often adjacent to chitinase catalytic domains, but this domain is also present in non-catalytic proteins either singly or as multiple repeats. CBM18 domains may be involved in targeting the degradative domains to particular carbohydrates, or may enhance catalysis by ensuring retention of the substrate between catalytic cycles.

About half of the genes with CAZy domains in the two *Leptosphaeria* species did not have a signal peptide and thus were not predicted as being secreted. This may be due to incorrect annotation at the 5’ end of genes, genes incorrectly merged during annotation, or a false negative result from SignalP. Pectate lyases were the most frequently secreted CAZy (100% for Lbc, 89% for Lmb), while GTs were the least frequently secreted (11% for Lbc, 12% for Lmb). This is consistent with the role of GTs in synthesising carbohydrates for the fungal cell wall, and their membrane or intracellular location.

**Figure 4:**
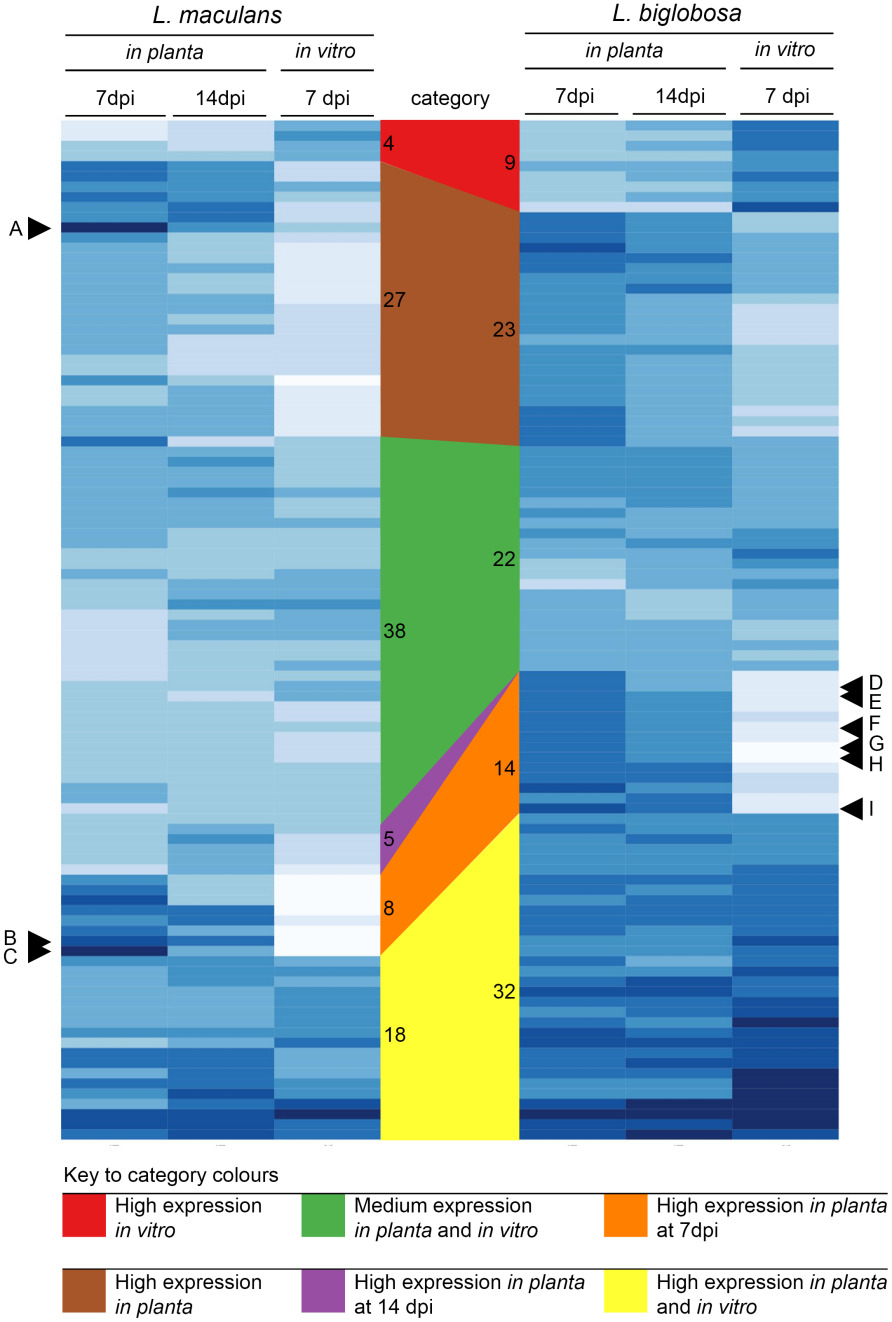
Expression profiles of secreted CAZys of *L. maculans* ‘brassicae’ (Lmb), *L. biglobosa *‘canadensis’ (Lbc) *in planta* and *in vitro*. The top 100 genes expressed across the three treatments (7, 14 dpi and *in vitro*) and predicted to be secreted and to contain a CAZy domain (CBM, GH, PL, or AA) were selected. Quantile-normalisation was applied and log10-transformed FPKM values were graphed. The intensity of blue shading is proportional to the expression level. The gene order is based on a dendrogram created from a Euclidean similarity matrix with average group distance. Letters with a triangle point to genes that were amongst the top 20 most highly expressed genes *in vitro* or *in planta* (Tables 2, 3, 4, or 5). A/Lema_P102640.1 (Lm2LysM); B/Lema_P013700.1 (Glycoside hydrolase family 7); C/Lema_P070100.1 (Lm5LysM); D/Lb_j154_P005286 (pectate lyase); E/Lb_j154_P009204 (cellulase); F/Lb_j154_P001246 (Glycosyl hydrolase family 43); G/Lb_j154_P001652 (cellulase); H/Lb_j154_P009247 (Glycosyl hydrolases family 12); I/Lb_j154_P004093 Glycosyl hydrolases family 39). Categories of gene expression (High expression *in vitro*, High expression *in planta*, Medium expression *in planta* and *in vitro*, High expression *in planta* at 14 dpi, High expression *in planta* at 7dpi, High expression *in planta* and *in vitro*) were manually assigned and indicated by coloured polygons linking the genes in each category across Lmb and Lbc; the number of genes in each category is indicated on the vertical sides of the polygon.

We then examined the RNA-seq data to determine expression levels of CAZys of Lbc and Lmb during infection of *B. napus* cotyledons (Table 7, Table S7 in file S2). The expression profiles of the top 100 most highly expressed (summed FPKM values at 7 dpi, 14 dpi, and *in vitro*) secreted CAZy genes are presented in Figure 4. At 7 dpi, Lmb had a high level of expression of genes with CBM domains, particularly CBM50/LysM (for instance, genes A and C in Figure 4); this level decreased by more than 50% by 14 dpi and was even lower during growth *in vitro*.

**Table 7:** Expression of CAZy genes in *L. maculans* ‘brassicae’ and *L. biglobosa* ‘canadensis.’

	*L. maculans*			*L. biglobosa*		
CAZy class	7dpi *in planta* (FPKM)	14dpi *in planta* (FPKM)	i*n vitro* (FPKM)	7dpi *in planta* (FPKM)	14dpi *in planta* (FPKM)	*in vitro* (FPKM)
AA	86.5	109.9	117.3	104.5	89.1	159.7
CBM	508.7	203.6	158.5	163.7	174.3	341.4
CE	78.1	69.5	63.6	78.7	55.8	99.9
GH	110.2	122.3	88.1	122.7	81.3	125.5
GT	54.8	60.9	57.1	46.3	50.0	112.3
PL	56.3	23.9	6.9	121.3	37.7	29.4

RNA-seq derived gene expression values were mapped onto the identified CAZy domains for Lmb and Lbc. Average expression levels (FPKM) were then calculated for each major class of CAZy.

Genes with CBM domains were also among the most highly expressed CAZy genes of Lbc *in planta*, but the overall level of expression was much lower than that of Lmb genes, at the same time point. To reveal the most *in planta* specific CAZy classes, expression ratios between *in planta* and *in vitro* growth were calculated for each CAZy class (Table S9 in file S2). For Lmb, the CBM50 (chitin-binding) domain was highly upregulated *in planta* at 7 dpi, whereas at the same time point Lbc upregulated CBM6 (cellulose binding) domains more highly (Table S9 in file S2). The *in planta* upregulated RNA-seq expression profile of the CBM50-containing genes of Lmb was validated by quantitative RT-PCR (Figure S2 in file S1). These experiments showed peak expression of each LysM gene at 7 dpi *in planta* and much lower expression levels *in vitro*. At 3 dpi, LysM gene expression was higher than *in vitro* levels, but lower than levels at 7 dpi. For the CBM18-containing genes, expression was varied, with >3 orders of magnitude between the highest and lowest expressed genes. *L. biglobosa* ‘canadensis’ had generally higher expression compared to that of Lmb (Table S8 in file S2). For Lbc, the average expression value of all CBM genes was highest during *in vitro* growth, which was due to high expression of several genes that had both CBMs and hydrolytic CAZY domains (Table 7). Such genes included Lb_j154_P004089, which has three CBM18 and one CE4 domains (Table S8 in file S2).

Polysaccharide lyases (PL) were expressed highly at 7 dpi, and expression decreased by 14 dpi in both species. At 7 dpi, the expression levels of PLs in Lbc were twice those of Lmb. Expression levels then dropped by 75% at 14 dpi and were low during growth *in vitro*. *L. biglobosa* ‘canadensis’ had higher levels of expression of degrading CAZys such as AAs, GHs and PLs (genes D,E,F,G,H and I; Figure 4) at 7 dpi, than at 14 dpi, when lesion growth had slowed. The PL3 class (pectate lyase) was highly upregulated by Lbc at 7 dpi (Table S9 in file S2), and was also the most upregulated PL class in Lmb. This may be a reflection of the abundance of pectin in the cotyledon.

Glycosyl transferase (GT) -containing genes were expressed at similar levels in all conditions in both *Leptosphaeria* species, except for a two-fold increase in Lbc grown *in vitro* compared to *in planta* (Table 7). The class GT21, which encodes biosynthetic enzymes that glycosylate lipids, was the most *in planta* upregulated class of glycosyltransferases. Of the carbohydrate esterase enzymes, CE8 (pectin methylesterase) and CE12 (pectin acetylesterase) were the most *in planta* upregulated classes for Lbc and Lmb, respectively (Table S9 in file S2).

At 14 dpi, of the six CAZy classes, expression of only PLs was higher in Lbc than in Lmb. By 14 dpi, expression of CBM and PL-containing genes by Lmb had decreased, but expression of genes with AA and GH domains had increased. *In vitro* growth was characterised by low CBM expression and very low PL expression (Table 7). The CAZy expression profile for Lmb *in planta* may reflect early avoidance of chitin- triggered immunity via high expression of CBMs such as LysM genes, whilst the fungus feeds from soluble pectin via PL genes. At 14 dpi elevated expression of the oxidative AA class and the hydrolytic GH class may facilitate degradation of lignin and cellulose in dead cells.

In many fungi, expression of cell wall degrading enzymes CAZys is regulated by carbon catabolite repression, a global mechanism that ensures readily assimilated carbon sources such as glucose are preferentially used. A Lmb homologue of the *Saccharomyces cerevisiae* sucrose non-fermenting protein kinase1 *(SNF1)* carbon catabolite regulator has been recently shown to regulate expression of several CAZy genes encoding pectate lyases, beta-1,3-glucanase, and a glucosidase [45]. These CAZy genes were upregulated four days after inoculation of canola cotyledons with a wild type strain of Lmb; whereas an LmSNF1 knockout strain had significantly reduced expression of these CAZys during growth on pectin *in vitro*. We recorded similar upregulation of the CAZy genes encoding two pectate lyases and the carbohydrate esterase *in planta* at 7 dpi, but not significant upregulation of the chitin deacetylase. This latter difference could be due to differing time points at which tissue was analysed and the more robust normalisation used in an RNA-seq analysis compared to a qPCR method.

In both Lmb and Lbc, the top 5% most highly expressed CAZy genes accounted for approximately 50% of the sum total CAZy expression (FPKM) at each growth stage (7 and 14 dpi and *in vitro* growth) that was analysed (data not shown). This suggests that the vast majority of CAZys are expressed at low levels or only turned on at specific times in the fungal life cycle. This phenomenon has been reported previously for *Z. tritici,* which differentially expresses CAZy genes, such as cutinases (CE5 subclass) according to biotrophic, necrotrophic or saprotrophic stages of growth [44]. The hemi-biotroph *Colletotrichum higginsianum* upregulates expression of effectors and secondary metabolite biosynthetic enzymes both before penetration and during biotrophic growth on *Arabidopsis*. *C. higginsaneum* then upregulates hydrolases and transporters at a later stage, each wave delivered according to the stage of pathogenic transition [46]. This again matches our observation that *L. biglobosa* lacks or only has a short biotrophic stage and expresses hydrolases earlier than Lmb does. CAZy genes of the two *Leptosphaeria* species that have low expression during development of cotyledonary lesions might be expressed more highly in another growth situation, such as earlier in penetration of the tissue, or during colonisation of petiole, stem, or during saprophytic growth on woody stubble of *B. napus*.

### Peptidase distribution and expression

Since the two *Leptosphaeria* species grow between the plant cells in the apoplast, protein from the plant cell wall is a potential source of nitrogen for nutrition. A trypsin-like peptidase was highly expressed during necrotic stages of infection for both Lmb (14 dpi) and Lbc (7 dpi) (Table 2, Table 3), therefore we characterised the peptidase content of both *Leptosphaeria* genomes and compared them to four other Dothideomycete plant pathogens.

The MEROPS peptidase classifications categorises peptidases by their catalytic nucleophile [37]. For example, aspartic (A), cysteine (C), serine (S), threonine (T) peptidases are named for the corresponding catalytic residue. Exceptions are for metallo (M) peptidases, which use a coordinated metal ion, and the classifications for unknown activities (U) and peptidase inhibitors (I). Peptidase composition and number was very similar for Lmb, Lbc and *A. brassicicola* (Table 8). Metallo- and serine-peptidase genes comprised almost 70% of the total peptidases. The next most abundant classes were the cysteine, threonine and aspartic peptidases, in that order. *L. biglobosa* ‘canadensis’ had seven C56 cysteine peptidases (7) whilst Lmb only had three. C56 peptidases act on peptides <20 amino acids, presumably requiring prior digestion of the substrate by another peptidase. *P. tritici-repentis* and *S. nodorum* had more peptidases in the metallo and serine classes than Lmb or Lbc. The hemi-biotroph *Z. tritici* had a larger complement of serine and aspartic peptidases than the two *Leptosphaeria* species did.

**Table 8:** Distribution of peptidase domains in six Dothideomycete genomes.

Peptidase type	A	C	G	I	M	S	T	U	Total
Species (Lifestyle)									
*Leptosphaeria biglobosa* (necrotroph)	18	73	1	8	144	145	23	1	413
*Leptosphaeria maculans* (hemibiotroph)	16	78	1	10	140	140	25	1	411
*Alternaria brassicicola* (necrotroph)	17	77	1	6	146	163	23	1	434
*Stagonospora nodorum* (necrotroph)	23	80	0	7	151	192	23	1	477
*Pyrenophora tritici-repentis* (necrotroph)	22	78	1	9	155	163	22	1	451
*Zymoseptoria tritici* (hemibiotroph)	33	67	4	8	130	191	23	1	457

Peptidase domains are classified according to the catalytic type, or inhibitor activity. Peptidase types are Aspartic (A), Cysteine (C), Glutamic (G), Inhibitor, Mettallo-(M), Serine (S), Threonine (T), Unknown (U).

In general, the expression levels of peptidase at 7 and 14 dpi *in planta* were similar in Lmb and Lbc (Pearson correlation coefficient of 0.76 for Lmb IBCN18 and 0.85 for Lbc J154), with a few notable exceptions. A trypsin-like serine peptidase (MEROPS S01A) was the most highly *in planta* up-regulated peptidase (500-fold higher at 7 dpi *in planta* than *in vitro)* in both Lbc (Lb_j154_P004138) and Lmb (Lema_P082270.1) (Table 2, Table S10, Table S11 in file S2). Its role in disease is unknown. The MEROPS S01A “type” peptidase is bovine chymotrypsin but fungal homologs are usually described as “trypsin-like” because they are similar to both bovine trypsin, and trypsin from *Streptococcus bacteria* [47]. The *in vitro* growth condition resulted in a more distinct expression profile of peptidases, producing low correlation co-efficient values (0.35 and 0.38) between the *in vitro* and 7 dpi *in planta* conditions, for Lbc and Lmb, respectively. Four Lmb peptidases had moderate levels of expression at 7 dpi and were down-regulated during growth *in vitro*, in a similar manner to the trypsin homologs. Two genes, Lema_P058000.1 and Lema_P031600.1, were most similar to C56 cysteine peptidases. The other two genes, Lema_P044030.1 and Lema_P044810.1, were most similar to the S33 serine peptidases, which typically release an N-terminal proline from a peptide substrate. These peptidases may target hydroxyproline-rich glycoproteins such as extensins, which are components of the plant cell wall [48]. Average CAZy class expression for the peptidases showed Lbc expressed its peptidase inhibitors more highly than Lmb did across all treatments (Table S11 in file S2). Glutamyl peptidases were expressed at very low levels in both fungi, and threonine peptidases had the highest average expression of all peptidase classes.

### Expression of Brassica genes

**Figure 5:**
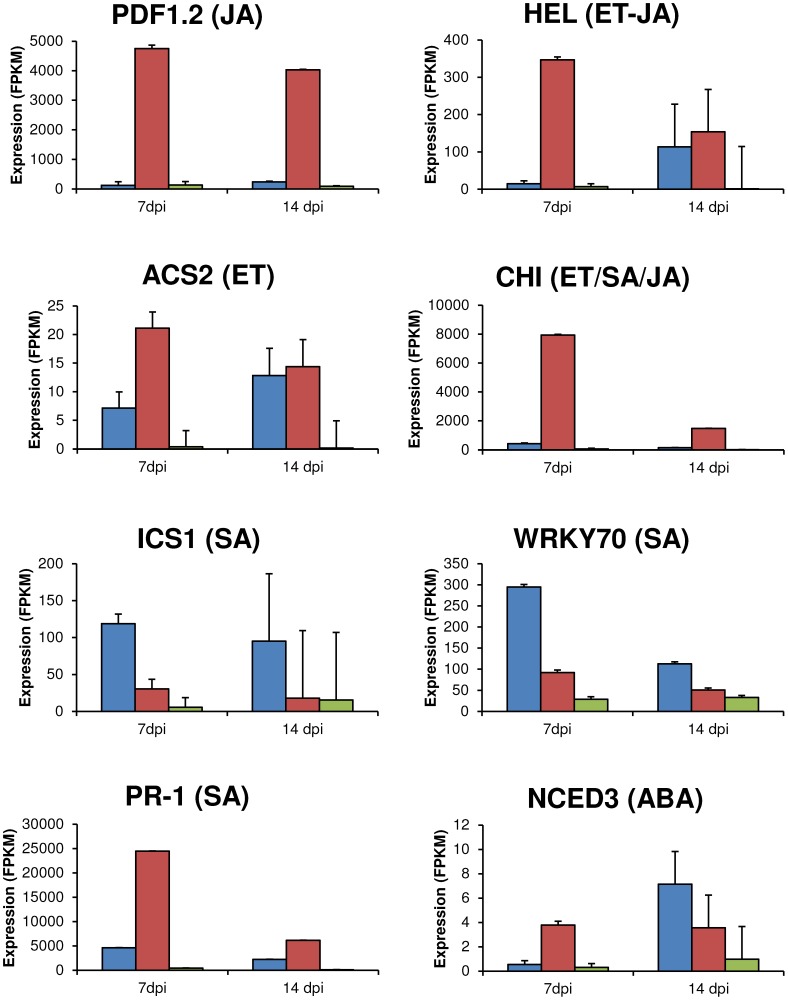
Expression of key defence genes of *B. napus* at 7 or 14 dpi with either *L. maculans* ‘brassicae’ (Lmb) or *L. biglobosa *‘canadensis’ (Lbc). RNA-seq data for eight key Brassica defence genes were determined at 7 and 14 dpi. Average expression (FPKM) after infection by *L. maculans* (blue), *L. biglobosa* (red), and mock inoculum (green) is plotted. Error bars indicate the Cufflinks 95% confidence interval for each FPKM value. The genes assayed were 9-cis-epoxycarotenoid dioxygenase 3 (NCED3), 1-amino-cyclopropane-1-carboxylate synthase 2 (ACS2), chitinase (CHI), hevein-like protein (HEL), isochorismate synthase 1 (ICS1), Pathogenesis related protein 1 (PR-1), WRKY transcription factor 70 (WRKY70), and plant defensin 1 (PDF1.2). Each gene was also classified according to hormone(s) abscisic acid (ABA), ethylene (ET), jasmonic acid (JA) and salicylic acid (SA) that induced higher expression.

The infection stage in a plant-pathogen interaction is often reflected in expression of key genes implicated in host defence signalling pathways. The expression levels for eight defence reporter genes of *B. napus* were determined during invasion by Lmb and Lbc (Figure 5). In general these genes were more highly expressed at 7 dpi during infection by Lbc than by Lmb. During Lbc infection, reporter genes for ethylene signalling (ACS2, CHI, and HEL) were expressed 50- to 138-fold higher than in mock-infected controls at 7 dpi, while Lmb infection resulted in only 2–19 fold induction of these genes. *L. maculans* ‘brassicae’ triggered a higher expression level of a key salicylic acid reporters, ICS1 and WRKY70, but another salicylic acid reporter gene, PR-1, was not as up regulated compared to Lbc. The salicylic acid reporter gene, PR-1, but not ICS1, were similarly highly induced at 7 dpi in Lbc. The jasmonic acid reporter gene PDF1.2 was induced highly by Lbc infection, but not by Lmb. The upregulation of these genes reflected that jasmonic acid and salicylic acid defence pathways were induced by Lbc, in a pattern consistent with the timing of necrosis.

Sasek et al (2012) showed that interactions of *B. napus* and Lmb, involving the recognition of *AvrLm1* (or the mutant allele, *avrLm1*) and corresponding resistance gene, *Rlm1*, salicylic acid biosynthesis and transcription of SA-associated genes (ICS1, WRKY70 and PR-1) increased as early as 3 dpi. Expression of HEL and CHI, genes involved in ethylene signalling, increased at 7 dpi [49], [50]. Although these genes were upregulated during the susceptible response compared to the uninoculated controls, they were much more highly expressed during a resistance response.

**Figure 6:**
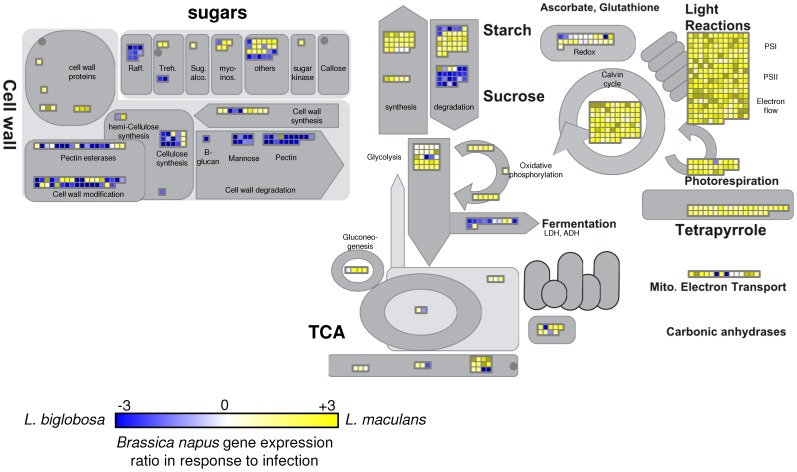
Transcription of *B. napus* genes involved in metabolic processes including photosynthesis seven days after inoculation with *L. maculans* ‘brassicae’ (Lmb) or *L. biglobosa *‘canadensis’ (Lbc). RNA-seq gene expression values for a *B. napus* unigene set [27] were used to calculate a ratio of expression values (log2) for *B. napus* genes after infection by Lmb or Lbc. Ratios were plotted on major metabolic pathways with Mapman software [39]. A yellow square indicates a *B. napus* gene that is expressed more highly during Lmb infection, while a blue square indicates a *B. napus* gene with higher expression during Lbc infection. An expression ratio close to zero is shown with a white square and indicates equivalent expression during infection by either pathogen. Only genes with expression values greater than 10 FPKM were included. Abbreviations: LDH, lactate dehydrogenase; ADH, Alcohol dehydrogenases, TCA, tricarboxylic acid cycle; raff, raffinose; Treh, trehalose; PSI, photosystem one; PSII, photosystem two; ABA, abscisic acid; ET, Ethylene; SA, salicylic acid; JA, jasmonic acid.

The increased level of expression of *B. napus* genes involved in ethylene, jasmonic acid and salicylic acid signalling during Lbc infections prompted further examination of biochemical pathways during infection. A ratio of expression values of *B. napus* genes at 7 days after infection with Lmb compared to Lbc was calculated and analysed by MapMan software to identify pathways co-ordinately responding to early infection of the cotyledon (Figure 6, Table 9). The major difference in host response to infection by the two fungi was in genes with photosynthesis-associated activities. *L. biglobosa* ‘canadensis’ infection resulted in massive down-regulation of genes involved in photosynthesis (e.g. PSI, PSII), electron transport, photorespiration and chlorophyll (tetrapyrrole) biosynthesis compared to that in Lmb. Similarly, expression of sucrose and starch biosynthesis genes was reduced, possibly as a flow-on from lack of photosynthesis. Levels of sucrose degradation genes were higher during Lbc than during Lmb infection, perhaps due to decreased amounts of photosynthate. Biosynthetic genes for raffinose, a monosaccharide osmoprotectant, were induced by Lbc, perhaps due to water stress. In contrast, genes involved in starch metabolism (both synthesis and degradation) were transcribed at high levels during Lmb infection at 7 dpi. The cell wall remodelling genes of *B. napus* that modify β-glucans, mannans and pectin were more highly expressed during infection by Lbc than by Lmb [51]. Extensive up regulation of host cell wall remodelling genes occurred at 7 dpi as the necrotic lesion was formed by Lbc, while Lmb infection had much less impact on transcription of these genes. A cohort of genes associated with secondary metabolism in *B. napus* was also plotted using Mapman on the same dataset (Figure S3 in file S1). Isoflavone reductase genes associated with isoflavonoid biosynthesis, were more highly expressed during Lbc infection, while genes associated with carotenoid metabolism (phytoene dehydrogenase, zeta-carotene desaturase, lycopene cyclases and violaxanthin de-epoxidase) were expressed more highly during Lmb infection. These observations are in agreement with the increased necrosis observed during Lbc infection at the early stages of infection. As well as increased expression of secondary metabolism genes during Lbc infection, peroxidase, nitrilase, and cytochrome P450 genes were consistently upregulated by *B. napus* at 7 dpi (Figure S3 in file S1). Twenty four general peroxidases were upregulated suggesting a strong oxidative burst was deployed during infection by Lbc. Forty two cytochrome 450 genes, 15 oxidase genes and 34 nitrilase genes were upregulated by Lbc infection, which also suggest a strong activation of secondary metabolism.

**Table 9:** Top 20 functional categories that are significantly regulated in *B. napus* in response to infection by *L. maculans* ‘brassicae’ compared to infection by *L. biglobosa* ‘canadensis.’

Mapman Function Category	Mapman Function Description	Number of genes	p-value
1	Photosynthesis	313	0.00E+00
1.1	Photosynthesis, light reactions	210	0.00E+00
1.1.1	Photosynthesis, light reactions, photosystem II	105	0.00E+00
26	Miscellaneous	365	4.42E-25
10	Cell wall	116	4.31E-12
1.1.2	Photosynthesis lightreaction photosystem I	44	5.06E-11
1.1.1.2	Photosynthesis lightreaction.photosystem II, PSII polypeptide subunits	63	5.06E-11
1.3	Photosynthesis calvin cycle	76	2.19E-10
1.1.1.1	PS lightreactions photosystem II, LHC-II	42	3.08E-10
1.1.2.2	PS lightreactions photosystem I, PSI polypeptide subunits	39	3.56E-10
20.1	Stress, biotic	123	1.98E-09
10.6	Cell wall, degradation	28	3.88E-09
26.12	Misc peroxidases	26	1.07E-08
26.8	Misc nitrilases, nitrile lyases, berberine bridge enzymes, reticuline oxidases, troponine reductases	42	3.72E-08
26.10	Misc cytochrome P450	46	1.93E-07
26.3	Misc gluco-, galacto- and mannosidases	42	1.35E-06
10.6.3	Cell wall degradation, pectate lyases and polygalacturonases	17	1.74E-05
35	Not assigned	1969	6.69E-05
35.2	Not assigned, unknown	1969	6.69E-05
17	Hormone metabolism	107	8.21E-05
2.2.1	Major CHO metabolism, degradation sucrose	22	6.47E-04
20.1.7.6	Stress biotic, PR-proteins, proteinase inhibitors	12	6.63E-04
1.2	Photosynthesis, photorespiration	27	7.32E-04
17.5	Hormone metabolism.ethylene	30	7.77E-04
34	Transport	208	8.58E-04
33	Development	107	1.62E-03
26.9	Misc glutathione-S-transferases	32	1.92E-03
10.8	Cell wall pectin esterases	18	1.92E-03
17.5.1	Hormone metabolism, ethylene synthesis-degradation	21	1.92E-03
20.1.7.6.1	Stress biotic, PR-proteins, proteinase inhibitors, trypsin inhibitor	11	1.92E-03
1.1.3	Photosynthesis, light reactions, cytochrome b6/f	8	2.25E-03
10.8.1	Cell wall,pectin esterases, PME	13	2.35E-03
31.4	Cell vesicle transport	32	2.41E-03
1.3.6	Photosynthesis, calvin cycle,aldolase	13	2.49E-03
1.1.4	Photosynthesis, lightreaction, ATP synthase	15	2.55E-03
29.5.3	Protein degradation, cysteine protease	18	3.11E-03
29.5.9	Protein degradation, AAA type	15	3.41E-03
2.2.1.3	Major CHO metabolism, degradation, sucrose invertases	13	4.18E-03
13.2.3	Amino acid metabolism, degradation, aspartate family	11	5.62E-03
1.1.5	Photosynthesis, light reaction, other electron carrier (oxidation/reduction)	18	5.77E-03

*B. napus* genes expressed during infection at 7 dpi were assigned to functional categories and overall pathway regulation was examined. The top 20 categories determined by MapMan analysis are listed. Abbreviations: PS (photosynthesis), LHC (light harvesting complex), CHO (carbohydrate), PR (pathogen response), PME (pectin methyl esterase), ATP (adenosine triphosphate). AAA type (ATPases Associated with diverse cellular Activities). P values indicate the likelihood of the observed pathway regulation being due to chance.

### Summary

The hemi-biotroph *L. maculans* ‘brassicae’ avoids triggering host defence during early infection. This is reflected by the finding that at seven days post inoculation, *L. maculans* ‘brassicae’ expresses a large number of genes with no known domains, many of them being small secreted proteins. One class of small-secreted protein-encoding genes that is highly expressed at this time is CBM50 (LysM) genes, which suppress chitin-triggered PAMP immunity and evade detection of the fungus by the plant. Also avirulence genes are highly upregulated; at seven days post-inoculation, two avirulence genes are amongst the top 20 most highly upregulated *L. maculans* ‘brassicae’ genes *in planta*. This pattern is consistent with the relatively asymptomatic growth phase of this fungus at seven days post-inoculation. In contrast, *L. biglobosa* ‘canadensis’ expresses a high number of cell wall degrading CAZy genes during the first seven days of infection, consistent with extensive necrosis and a high degree of activation of host defence signalling pathways.

## Supporting Information

File S1 **Supporting figures. Figure S1,** Classifications of CBM50 (LysM) domains in *L. maculans* ‘brassicae’, *L. biglobosa* ‘canadensis’, *Cladosporium fulvum* and *Zymoseptoria tritici.* LysM domains from Lmb, Lbc and *Z. tritici* (formerly *Mycosphaerella graminicola*) with high sequence similarity to ECP6 of *C. fulvum* were aligned using ClustalW. (A) Domains are numbered by proximity to N-terminus (#1 is closest). Residues are coloured by similarity (black: 100%, dark grey: 80–100%, light grey: 60–80%, white: less than 60%). B) Phylogram based on the amino acid alignment of LysM domains from panel A. Branch numbers show % bootstrap support and scale bar shows amino acid substitutions per site. LysM domains assigned to three Positions A, B and C based on sequence similarity to the *C. fulvum* ECP6 sequence. LysM domain organisation in ECP6-like predicted proteins in Lm, Lb, *Z. tritici* and *C. fulvum* are shown in panel C. Mg LysM genes are from *Z. tritici.* **Figure S2,** Expression of three LysM-containing genes of *L. maculans* ‘brassicae’ grown *in vitro* and *in planta.* Quantitative RT-PCR analysis was performed on RNA from Lmb isolate IBCN18 grown in 10% Campbells V8 juice (*in vitro*), and after infection of cotyledons of *B. napus* cv. Westar at 3, 7 and 14 days post inoculation (dpi). Expression levels of Lm2LysM (Lema_P102640.1), Lm4LysM (Lema_P025400.1), Lm5LysM (Lema_P070100.1), were normalised to those of gamma-actin (Lema_P099940.1). Error bars represent one standard error of the mean (n = 2–3 biological replicates). Asterisks indicate values significantly different from *in vitro* levels (p<0.05). **Figure S3,** Response of *B. napus* secondary metabolism and large enzyme families to infection by *L. maculans* ‘brassicae’ or *L. biglobosa* ‘canadensis.’RNA-seq gene expression values for a *B. napus* unigene set [27] were used to calculate a ratio of expression values (log2) for *B. napus* genes seven days after infection by Lmb or Lbc. Ratios were plotted on secondary metabolism gene groups using MapMan software on maps for ‘Secondary metabolism’, and ‘Large enzyme families’ maps [39]. A yellow square indicates a *B. napus* gene that is expressed more highly during Lmb infection, while a blue square indicates a *B. napus* gene with higher expression during Lbc infection. An expression ratio close to zero is shown with a white square and indicates equivalent expression during infection by either pathogen. Only genes with expression values greater than 10 FPKM were included. MVA is mevalonic acid.
(PDF)

File S2 **Supporting tables. Table S1,** Total number of RNA-seq reads aligned to reference genomes. **Table S2,** Oligonucleotide Primers. **Table S3,** Top 100 most highly upregulated *in planta* genes in *L. maculans* ‘brassicae’ at seven days post-inoculation. **Table S4,** Top 100 most highly upregulated *in planta* genes in *L. biglobosa ‘*canadensis’ at seven days post-inoculation. **Table S5,** Top 100 most highly upregulated *in planta* genes in *L. maculans* ‘brassicae’ at 14 days post-inoculation. **Table S6,** Top 100 most highly upregulated *in planta* genes in *L. biglobosa ‘*canadensis’ at 14 days post-inoculation. **Table S7,** Annotated CAZy domains of *L. maculans* and *L. biglobosa,* and their expression. **Table S8,** Multi-domain CBM18 genes of *L. maculans* ‘brassicae’ and *L. biglobosa* ‘canadensis’, and their expression. **Table S9,** The top 10 CAZy families of *L. maculans* ‘brassicae’ and *L. biglobosa* ‘canadensis’ based on expression ratio of *in planta* and *in vitro* growth at 7 dpi. **Table S10,** Annotated peptidases of *L. maculans* and *L. biglobosa,* and their expression. **Table S11,** Peptidase expression in *L. maculans* ‘brassicae’ and *L. biglobosa* ‘canadensis’.
(XLSX)
