# Supplementary material for: Genomes and Transcriptomes of Partners in Plant-Fungal- Interactions between Canola (Brassica napus) and Two Leptosphaeria Species
Source: PLoS One. 2014 Jul 28;9(7):e103098. doi: 10.1371/journal.pone.0103098 (PMC4113356; doi:10.1371/journal.pone.0103098)

Supporting data

Supporting text

CBM50 (LysM) genes in *L. maculans* 'brassicae' and *L. biglobosa* 'canadensis'.

Three LysM genes were identified in each Leptosphaeria genome, Lema_P070100.1, Lema_P025400.1 and Lema_P102640.1 in Lmb, and Lbj154_P004290, Lbj154_P000691 and Lbj154_P003458 in Lbc (Figure S1). Individual LysM domains were identified within each protein after alignment with CfECP6 from *Cladosporium fulvum*. Signal peptide sequences were identified using SignalP. The predicted proteins contained multiple cysteine residues. Genes with several LysM domains are hereafter referred to as 2LysM for a 2-domain gene, 3LysM for a 3-domain gene, etc. LysM domains were of a consistent length, with four residues conserved in all sequences, two flanking cysteines, an asparagine at position 33, and a proline at position 49. The LysM domain sequences were aligned using ClustalW and a BLOSUM cost matrix (Gap open 10, gap extend 0.1), then trimmed to the flanking cysteine residues.

A neighbour-joining phylogram was constructed from the alignment using Jules-Cantor genetic distance model and 50,000 bootstrap replicates (Figure S1 B). It revealed three broad groupings of domains, with sequence conservation primarily according to domain position in the mature protein rather than by the taxonomy of the organism. Lema_P070100.1 contained 5 LysM domains, which included three copies of the Position A type LysM domain. All other proteins contained one of each domain types A, B, and C. Lbc contained a gene with three LysM domains, similar to the CfECP6 gene, rather than to the Lmb gene, which had five LysM domains.

The RNA-seq data verified the manual annotation of these LysM genes. The expression of Lm5LysM (Lema_P070100.1), Lm2LysM (Lema_P102640.1) and Lm4LysM (Lema_P025400.1) during infection of cotyledons was determined independently by qRT-PCR (Figure S2) at 3, 7 and 14 dpi. Expression of all three Lmb genes was low at 3 dpi, but still higher than the expression *in vitro*. Lm5LysM and Lm2LysM were more highly expressed at 7 dpi than at 14 dpi, and had lower expression during growth *in vitro*. Lm4LysM was not as highly expressed *in planta*, but had low expression during growth *in vitro*. Lm2LysM had the highest expression *in vitro*.

Supplementary bibliography

Marshall R, Kombrink A, Motteram J, Loza-Reyes E, Lucas J, et al. (2011) Analysis of two *in planta* expressed LysM effector homologs from the fungus *Mycosphaerella graminicola* reveals novel functional properties and varying contributions to virulence on wheat. Plant Physiol. 2011 Jun;156(2):756-69.

Figure S1:

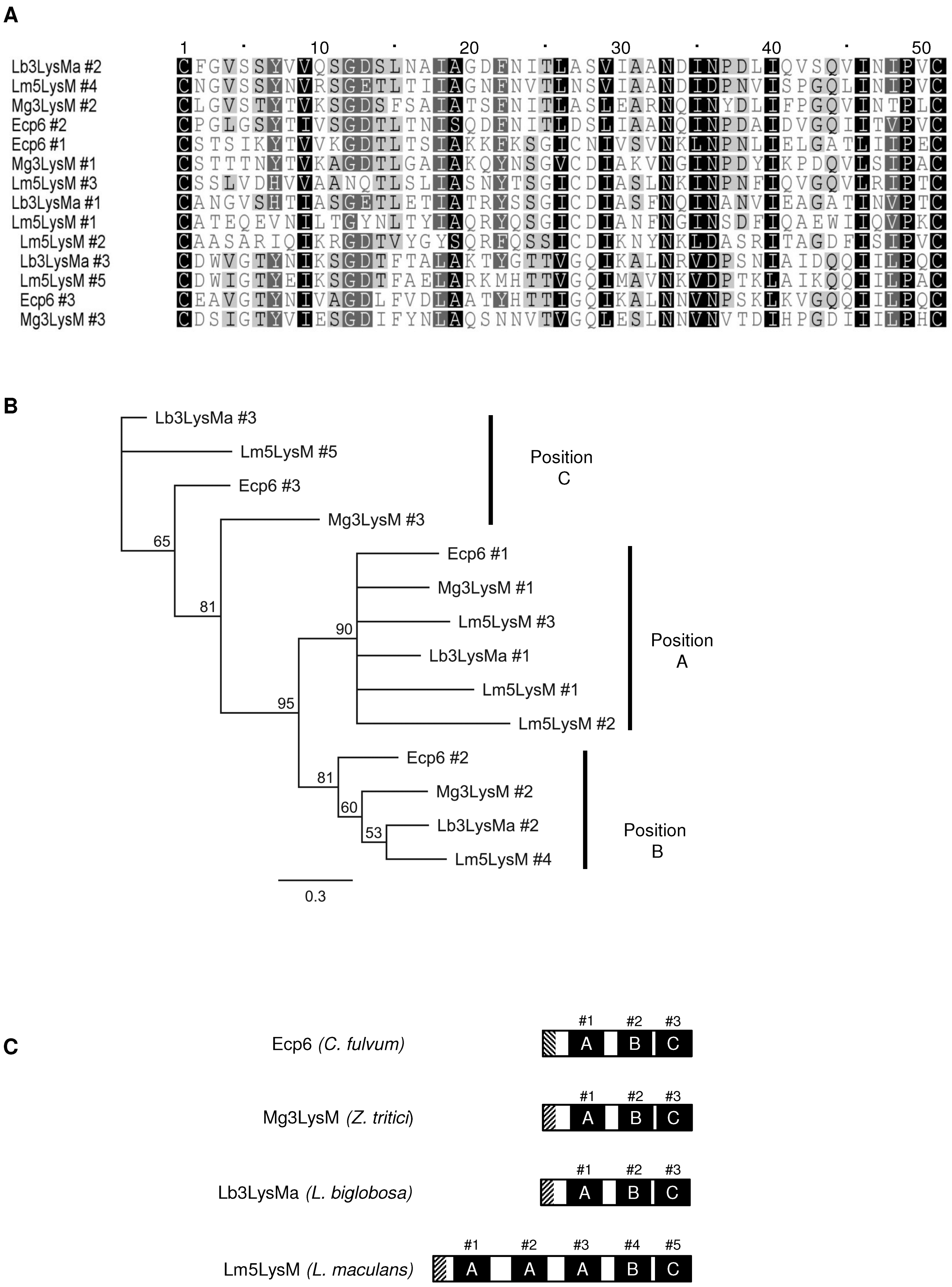
A
1 10 20 30 40 50
Lb3LysMa #2 CFGVSSYVVQSGDSLNAIAGDFNITLASVIAANDINPDLIQVSQVINIPVC
Lm5LysM #4 CNGVSSYNVRSGETLTIIAGNFNVTLNSVIAANDIDPNVISPGQLINIPVC
Mg3LysM #2 CLGVSTYTVKSGDSFSAIATSFNITLASLEARNQINYDLIFPGQVINTPLC
Ecp6 #2 CPGLGSYTIVSGDTLTNISQDFNITLDSLIAANQINPDAIDVGQIITVPVC
Ecp6 #1 CSTSIKYTVVKGDTLTSIAKKFKSGICNIVSVNKLNPNLIELGATLIIPEC
Mg3LysM #1 CSTTTNYTVKAGDTLGAIAKQYNSGVCDIAKVNGINPDYIKPDQVLSIPAC
Lm5LysM #3 CSSLVDHVVAANQTLSLIASNYTSGICDIASLNKINPNFIQVGQVLRIPTC
Lb3LysMa #1 CANGVSHTIASGETLETIATRYSSGICDIASFNQINANVIEAGATINVPTC
Lm5LysM #1 CATEQEVNILTGYNLTYIAQRYQSGICDIANFNGINSDFIQAEWIIQVPKC
Lm5LysM #2 CAASARIQIKRGDTVYGYSQRFQSSICDIKNYNKLDASRITAGDFISIPVC
Lb3LysMa #3 CDWVGTYNIKSGDTFTALAKTYGTTVGQIKALNRVDPSNIAIDQQIILPQC
Lm5LysM #5 CDWIGTYEIKSGDTFAELARKMHTTVGQIMAVNKVDPTKLAIKQQIILPAC
Ecp6 #3 CEAVGTYNIVAGDLFVDLAATYHTTIGQIKALNNVNPSKLKVGQQIILPQC
Mg3LysM #3 CDSIGTYVIESGDIFYNLAQSNNVTVGQLESLNNVNVTDIHPGDIIILPHC
B
Lb3LysMa #3
Lm5LysM #5
Ecp6 #3
Mg3LysM #3
Position C
65
81
Ecp6 #1
Mg3LysM #1
Lm5LysM #3
Lb3LysMa #1
Lm5LysM #1
Lm5LysM #2
Position A
90
95
Ecp6 #2
Mg3LysM #2
Lb3LysMa #2
Lm5LysM #4
Position B
81
60
53
0.3
C
Ecp6 (C. fulvum)
#1 #2 #3
A B C
Mg3LysM (Z. tritici)
#1 #2 #3
A B C
Lb3LysMa (L. biglobosa)
#1 #2 #3
A B C
Lm5LysM (L. maculans)
#1 #2 #3 #4 #5
A A A B C

Figure S2:

A

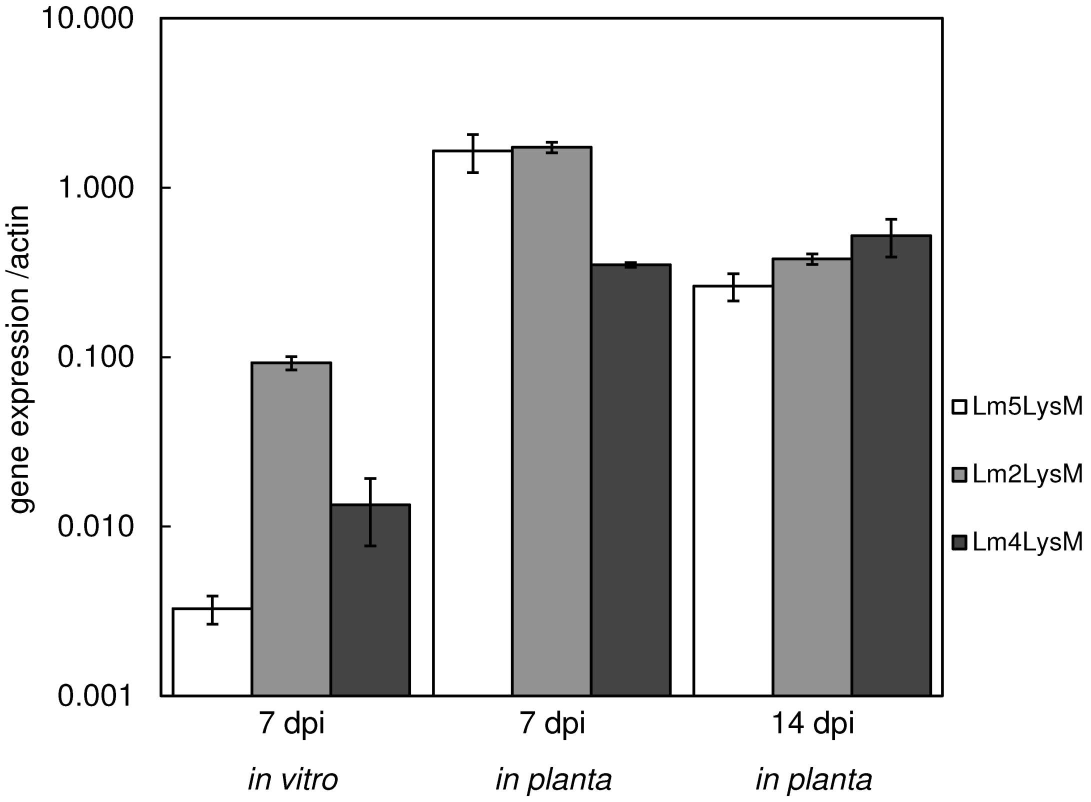
10.000
1.000
0.100
0.010
0.001
gene expression /actin
7 dpi
in vitro
7 dpi
in planta
14 dpi
in planta
Lm5LysM
Lm2LysM
Lm4LysM

B

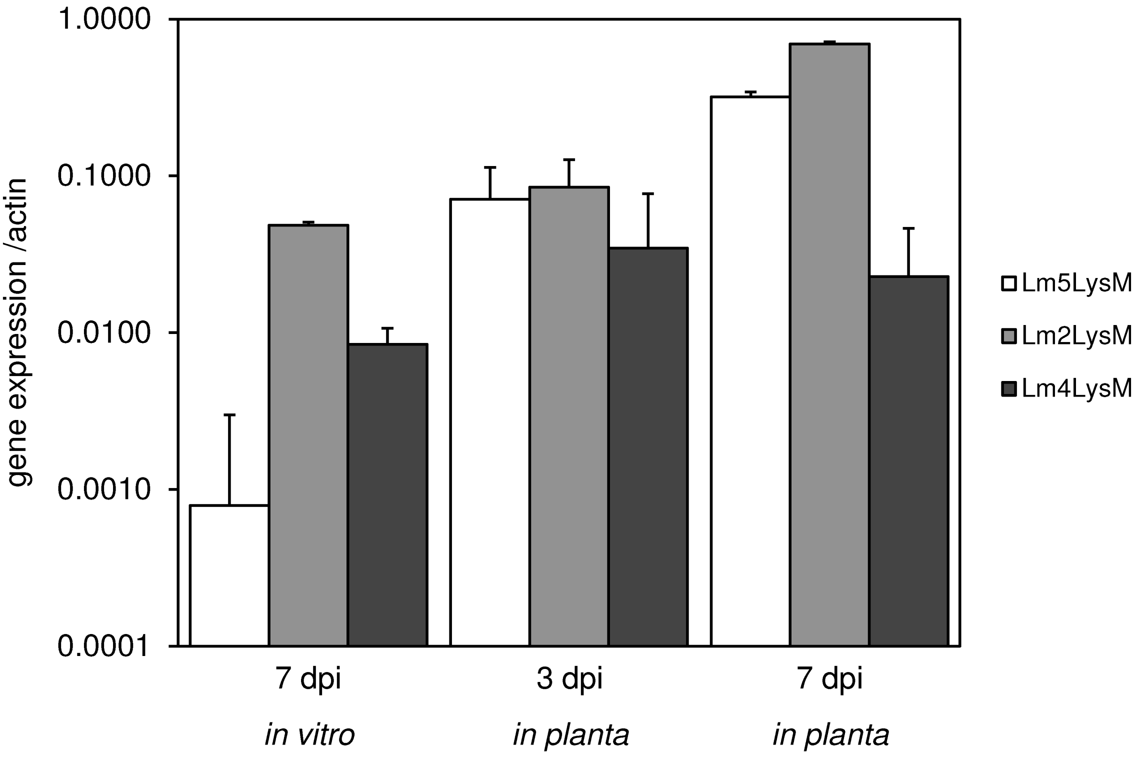
1.0000
0.1000
0.0100
0.0010
0.0001
gene expression /actin
7 dpi
in vitro
3 dpi
in planta
7 dpi
in planta
Lm5LysM
Lm2LysM
Lm4LysM

Figure S3: Response of *B. napus* secondary metabolism and large enzyme families to infection by *L. maculans* ‘brassicae ‘or *L. biglobosa* ‘canadensis’

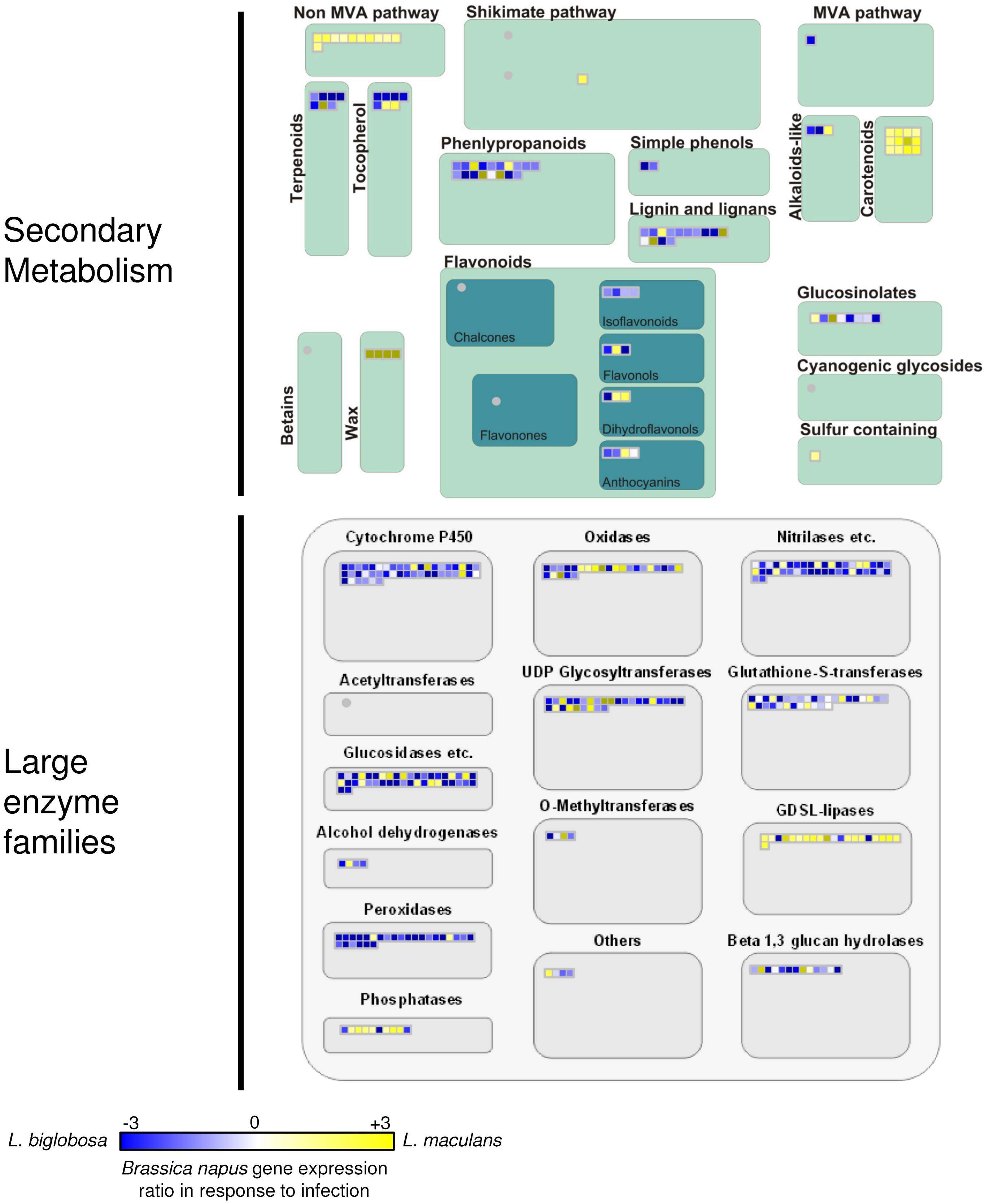

Supplement: File S1 — Supporting figures. Figure S1, Classifications of CBM50 (LysM) domains in L. maculans ‘brassicae’, L. biglobosa ‘canadensis’, Cladosporium fulvum and Zymoseptoria tritici. LysM domains from Lmb, Lbc and Z. tritici (formerly Mycosphaerella graminicola) with high sequence similarity to ECP6 of C. fulvum were aligned using ClustalW. (A) Domains are numbered by proximity to N-terminus (#1 is closest). Residues are coloured by similarity (black: 100%, dark grey: 80–100%, light grey: 60–80%, white: less than 60%). B) Phylogram based on the amino acid alignment of LysM domains from panel A. Branch numbers show % bootstrap support and scale bar shows amino acid substitutions per site. LysM domains assigned to three Positions A, B and C based on sequence similarity to the C. fulvum ECP6 sequence. LysM domain organisation in ECP6-like predicted proteins in Lm, Lb, Z. tritici and C. fulvum are shown in panel C. Mg LysM genes are from Z. tritici. Figure S2, Expression of three LysM-containing genes of L. maculans ‘brassicae’ grown in vitro and in planta. Quantitative RT-PCR analysis was performed on RNA from Lmb isolate IBCN18 grown in 10% Campbells V8 juice (in vitro), and after infection of cotyledons of B. napus cv. Westar at 3, 7 and 14 days post inoculation (dpi). Expression levels of Lm2LysM (Lema_P102640.1), Lm4LysM (Lema_P025400.1), Lm5LysM (Lema_P070100.1), were normalised to those of gamma-actin (Lema_P099940.1). Error bars represent one standard error of the mean (n = 2–3 biological replicates). Asterisks indicate values significantly different from in vitro levels (p<0.05). Figure S3, Response of B. napus secondary metabolism and large enzyme families to infection by L. maculans ‘brassicae’ or L. biglobosa ‘canadensis.’RNA-seq gene expression values for a B. napus unigene set [27] were used to calculate a ratio of expression values (log2) for B. napus genes seven days after infection by Lmb or Lbc. Ratios were plotted on secondary metabolism gene groups using MapMan [file pone.0103098.s001.pdf]
